# Simultaneous assessment of myocardial perfusion and adrenergic innervation in patients with heart failure by low-dose dual-isotope CZT SPECT imaging

**DOI:** 10.1007/s12350-022-02951-4

**Published:** 2022-04-04

**Authors:** Roberta Assante, Adriana D’Antonio, Teresa Mannarino, Carmela Nappi, Valeria Gaudieri, Emilia Zampella, Pietro Buongiorno, Valeria Cantoni, Roberta Green, Nicola Frega, Hein J. Verberne, Mario Petretta, Alberto Cuocolo, Wanda Acampa

**Affiliations:** 1 Department of Advanced Biomedical Sciences, University of Naples Federico II, Via Pansini 5, 80131 Naples, Italy; 2 Department of Radiology and Nuclear Medicine, Amsterdam University Medical Centers, location AMC, University of Amsterdam, Amsterdam, The Netherlands; 3 IRCCS Synlab SDN, Naples, Italy

**Keywords:** Heart failure, Innervation tracers, Perfusion agents, SPECT

## Abstract

**Background:**

In patients with heart failure (HF) sequential imaging studies have demonstrated a relationship between myocardial perfusion and adrenergic innervation. We evaluated the feasibility of a simultaneous low-dose dual-isotope ^123^I/^99m^Tc-acquisition protocol using a cadmium-zinc-telluride (CZT) single-photon emission computed tomography (SPECT) camera.

**Methods and results:**

Thirty-six patients with HF underwent simultaneous low-dose ^123^I-metaiodobenzylguanidine (MIBG)/^99m^Tc-sestamibi gated CZT-SPECT cardiac imaging. Perfusion and innervation total defect sizes and perfusion/innervation mismatch size (defined by ^123^I-MIBG defect size minus ^99m^Tc-sestamibi defect size) were expressed as percentages of the total left ventricular (LV) surface area. LV ejection fraction (EF) significantly correlated with perfusion defect size (*P* < .005), innervation defect size (*P* < .005), and early (*P* < .05) and late (*P* < .01) ^123^I-MIBG heart-to-mediastinum (H/M) ratio. In addition, late H/M ratio was independently associated with reduced LVEF (*P* < .05). Although there was a significant relationship (*P* < .001) between perfusion and innervation defect size, innervation defect size was larger than perfusion defect size (*P* < .001). At multivariable linear regression analysis, ^123^I-MIBG washout rate (WR) correlated with perfusion/innervation mismatch (*P* < .05).

**Conclusions:**

In patients with HF, a simultaneous low-dose dual-isotope ^123^I/^99m^Tc-acquisition protocol is feasible and could have important clinical implications.

**Supplementary Information:**

The online version contains supplementary material available at 10.1007/s12350-022-02951-4.

## Introduction

Cardiac ^123^I-metaiodobenzilguanidine (MIBG) imaging has a central role in the evaluation of cardiac sympathetic activity, and impairment of innervation status has been correlated to a poor prognosis in patients with heart failure (HF).^1^ Semiquantitative parameters of ^123^I-MIBG uptake, such as the heart-to-mediastinum (H/M) ratio and washout rate (WR), indicators of autonomic dysfunction, demonstrated prognostic value in patients with HF.^2^ Prior studies evaluating myocardial perfusion and adrenergic innervation with two separate single-photon emission computed tomography (SPECT) acquisition procedures demonstrated that quantification of perfusion/innervation mismatch provides information about the trigger zone as a prognostic factor of ventricular arrhythmia.^3^

The introduction of novel dedicated SPECT cameras with semiconductor cadmium–zinc–telluride (CZT) detectors has enabled significant improvements in spatial, temporal and energy resolution in the acquisition protocols by comparison to conventional Anger cameras.^4,5^ Phantom and clinical studies have shown good correlation between ^123^I-MIBG H/M ratio and WR obtained by CZT-SPECT and Anger cameras for single tracer studies.^6–8^ The increased energy resolution of CZT-SPECT allows a simultaneous assessment of myocardial perfusion and sympathetic innervation in a single-session, thereby reducing imaging time. Thus, when using a low-dose of ^123^I-MIBG and ^99m^Tc-labeled tracers, it is possible to considerably reduce radiation exposure.

Recently Blaire et al^9^ in a phantom study assessing perfusion (^99m^Tc) and innervation (^123^I) using two commercially available CZT-SPECT cameras demonstrated that a simultaneous dual-isotope (SDI) acquisition is feasible and provides perfectly registered functional images with a reduced imaging time. Prior studies have demonstrated correlation between the impairment of innervation, rest perfusion and mechanical dyssynchrony or diastolic function using sequential perfusion and innervation imaging.^10,11^ However, no studies are available on the quantitative evaluation of perfusion and adrenergic innervation in patients with HF using a simultaneous acquisition protocol by CZT-SPECT. The aim of our study was firstly to evaluate the feasibility of a simultaneous low-dose dual-isotope ^123^I/^99m^Tc-acquisition protocol using a CZT-SPECT camera. We also assessed the relationship between myocardial perfusion, adrenergic innervation and left ventricular (LV) function in patients with HF undergoing this protocol.

## Methods

### Patients

Thirty-six consecutive patients with HF referred to ^123^I-MIBG imaging to evaluate cardiac adrenergic innervation, underwent low-dose SDI imaging acquisition. Heart failure was defined as the presence of typical symptoms (e.g., breathlessness, ankle swelling, and fatigue) and/or evidence of structural and/or functional cardiac abnormality, with preserved (≥ 50%), mid-range (40%-49%) or reduced (< 40%) LV ejection fraction (EF).^12^ The exclusion criteria were atrial fibrillation, implanted pacemaker or prosthetic valve, iodine allergy, severe loss of renal function, symptomatic asthma and pregnancy. For each patient, demographic data and clinical characteristics were noted. Hypertension was defined as a blood pressure > 140/90 mmHg or the use of anti-hypertensive medication. Dyslipidemia was defined as total cholesterol level > 6.2 mmol/L or treatment with cholesterol lowering medication. Patients were classified as having diabetes if they were receiving treatment with oral hypoglycemic drugs or insulin. A patient was considered to have known coronary artery disease based on a history of myocardial infarction or coronary revascularization. Nineteen subjects undergoing ^123^I-MIBG scintigraphy to rule out disease of the adrenal medulla served as the control group. None of these subjects had a history of neurological or cardiac diseases. The study was approved by the local ethical committee (Protocol Number 110/17) and conformed to the Declaration of Helsinki on human research. Written informed consent was obtained from every patient after complete explanation of the protocol, its aim and potential risks.

### CZT-SPECT imaging

**Figure 1:**
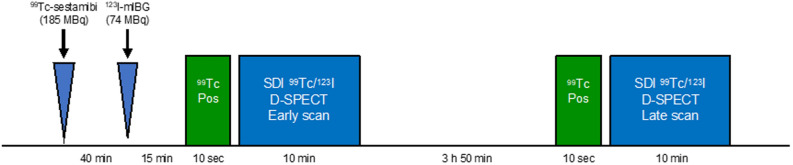
Simultaneous dual-isotope (SDI) low-dose ^99m^Tc-sestamibi/^123^I-MIBG imaging protocol. *Pos*, positioning

All patients underwent a low-dose SDI ^123^I-MIBG/^99m^Tc-sestamibi imaging protocol (Figure 1) using a cardiac dedicated CZT-SPECT camera (D-SPECT, Spectrum Dynamics, Caesarea, Israel). According to the study protocol, 185 MBq of ^99m^Tc-sestamibi were administered for rest myocardial perfusion imaging. After 40 minutes, 74 MBq of ^123^I-MIBG was administered over 1-2 minutes and 5 minutes later, a 10 second pre-scan acquisition using ^99m^Tc-window was performed to help with detector positioning, followed by a 10 minutes list mode SDI scan (early image). Similarly, 3 hours and 50 minutes after ^123^I-MIBG administration, a 10 minutes list mode SDI scan (late image) was performed.

Data were acquired by nine mobile CZT detector columns mounted vertically in 90° geometry (64 × 16 pixels, 120 projections per detector). List mode acquisition permits the retrospective selection of ^99m^Tc and ^123^I energy windows. The energy windows were approximately 8.5 % asymmetrical (− 7.5 to +4.5 keV) centered on 159 keV for ^123^I and 10 % asymmetrical (− 7 to +9 keV) centered on 140.5 keV for ^99m^Tc. Summed and gated projections were reconstructed with an iterative maximum likelihood expectation maximization algorithm using 7 and 4 iterations, respectively. No attenuation or scatter correction was performed.

### Imaging interpretation

Using the D-SPECT camera, the planar equivalent image (planogram) was obtained by projecting and summing all the elementary 2-D images that shared the same angle onto one large field of view virtual plane. On the ^99m^Tc-sestamibi images, a region of interest (ROI) was manually drawn on the left ventricle while size of the mediastinum ROI was determined automatically on the x and y dimensions and positioned manually, as previously reported.^6^ ROI were automatically copied from the ^99m^Tc-sestamibi images to the ^123^I-MIBG images using the software provided by the manufacturer. Once ROI were drawn, H/M ratios were calculated by the same nuclear cardiologists from early and delayed planar ^123^I-MIBG images, as previously reported.^13^ ^123^I-MIBG WR was also calculated using the following formula: [(early heart counts/pixel − early mediastinum counts/pixel) − (late heart counts/pixel decay-corrected − late mediastinum counts/pixel decay-corrected)]/(early heart counts/pixel − early mediastinum counts/pixel).

LV volumes, EF and perfusion and innervation defect scores were automatically calculated from gated ^99m^Tc-sestamibi images using a commercially available software (e-soft, 2.5, QGS/QPS, Cedars- Sinai Medical Center, Los Angeles, CA, USA). The myocardial perfusion and innervation images obtained at rest were scored using a 17-segment model of the left ventricle.^14^ Perfusion and innervation total defect sizes were expressed as the percentage of the total left ventricle surface area using as reference an internal normal database. Perfusion/innervation mismatch size was defined by ^123^I-MIBG defect size minus ^99m^Tc-sestamibi defect size, again expressed as a percentage of left ventricle surface area.

### Statistical analysis

Continuous variables were expressed as mean ± standard deviation (SD) and categorical data as frequencies or percentage. Comparison of continuous data between groups was performed using the two-sided Student’s *t* test and by one-way ANOVA followed by post hoc multiple comparisons with the Bonferroni correction. Evaluation of relationships between variables was performed using Spearman’s rank correlation analysis. Logistic regression analysis was performed to identify the predictors of reduced LVEF. Moreover, linear regression analysis was performed to identify the predictors of perfusion/innervation mismatch. For the multivariable analysis we only considered variables resulting statistically significant at univariable analysis. Statistical analysis was performed with Stata 15.1 software (StataCorp, College Station, Texas USA). A *P* value < .05 (two-sided) was considered statistically significant.

## Results

### Patient characteristics

**Figure 2:**
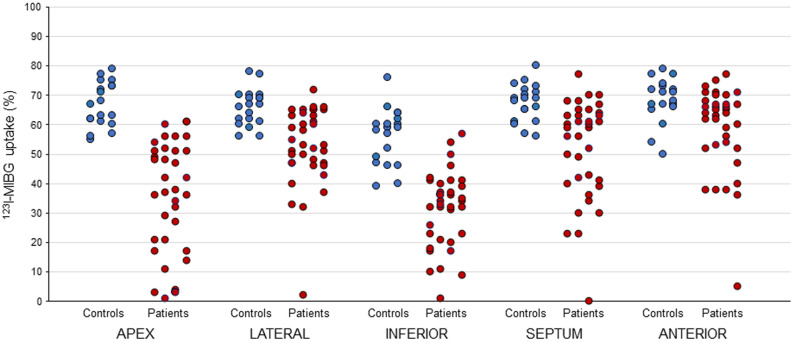
Individual regional ^123^I-MIBG uptake in controls (*n* = 19) and patients (n = 36)

Mean regional ^123^I-MIBG uptake in controls and patients is reported in Table 1. In controls, tracer uptake was reduced in the inferior region compared to the other regions (*P* < .05). In patients, both apex and inferior walls showed a lower ^123^I-MIBG uptake compared to the other regions (*P* < .01). To rule out that the ^123^I-MIBG uptake reduction in the inferior region in patients may reflect normal physiology more than pathological patterns, we compared individual uptake and found that in 26 (72%) patients tracer activity was lower than 2 SD of the mean values in control group (Figure 2).

**Table 1:** Mean regional ^123^I-MIBG uptake in controls and patients

	Apex	Lateral	Inferior	Septum	Anterior	*P* value
Controls (n = 19)	67 ± 7	66 ± 6	57 ± 10	67 ± 6	68 ± 7	.001
Patients (n = 36)	35 ± 19	53 ± 13	31 ± 13	52 ± 17	59 ± 15	.001

Values are expressed as mean ± SD

Of the 36 patients enrolled, 13 (36%) had a preserved or mid-range (48% ± 2%) LVEF and 23 (64%) a reduced (30% ± 6%) LVEF. The clinical and demographic characteristics of the patient population according to LVEF are described in Table 2. No significant differences between the two groups were observed. The myocardial perfusion and innervation parameters at SDI imaging are described in Table 3. Patients with reduced LVEF had higher values of perfusion and innervation defect sizes and WR (all *P* < .05) and lower values of early (*P* < .05) and late H/M ratio (*P* < .01). Conversely, the extent of perfusion/innervation mismatch was not different (*P* = .80) between patients with preserved or mid-range and reduced LVEF.

**Table 2:** Demographic data and clinical characteristics of study population according to left ventricular ejection fraction

	All patients (n = 36)	LVEF < 40% (n = 23)	LVEF ≥ 40% (n = 13)	*P* value
Age (years)	64 ± 9	65 ± 9	64 ± 9	.81
Male gender, n (%)	34 (94)	21 (91)	13 (100)	.27
Diabetes, n (%)	18 (50)	12 (52)	6 (46)	.73
Hypertension, n (%)	36 (100)	23 (100)	13 (100)	.10
Dyslipidemia, n (%)	32 (89)	21 (95)	11 (85)	.54
Smoking history, n (%)	11 (31)	5 (22)	6 (46)	.44
Family history of CAD, n (%)	14 (39)	11 (48)	3 (23)	.14
Prior myocardial infarction, n (%)	27 (75)	19 (83)	8 (61)	.16
Prior PCI, n (%)	25 (69)	16 (69)	9 (62)	.98
Symptoms, n (%)	21 (58)	12 (52)	9 (69)	.31
LV hypertrophy, n (%)	14 (39)	5 (22)	9 (69)	< .05

Values are expressed as mean value ± standard deviation or number (percentage) of subjects

*LVEF*, left ventricular ejection fraction; *CAD*, coronary artery disease; *PCI*, percutaneous coronary intervention

**Table 3:** Imaging findings of study population according to left ventricular ejection fraction

	All patients (n = 36)	LVEF < 40% (n = 23)	LVEF ≥ 40% (n = 13)	*P* value
Perfusion defect size (%)	17 ± 16	22 ± 17	9 ± 12	< .05
Innervation defect size (%)	29 ± 17	33 ± 17	21 ± 14	< .05
Perfusion/innervation mismatch area (%)	12 ± 14	11 ± 16	13 ± 10	.80
Early *H*/*M* ratio	1.69 ± 0.33	1.60 ± 0.32	1.84 ± 0.29	< .05
Late *H*/*M* ratio	1.65 ± 0.37	1.50 ± 0.29	1.93 ± 0.35	< .005
Washout rate (%)	28 ± 27	34 ± 21	16 ± 34	< .05

Values are expressed as mean value ± standard deviation

*LVEF*, left ventricular ejection fraction; *H/M*, heart-to-mediastinum

### Relationship between perfusion and innervation total defect sizes and LVEF

**Figure 3:**
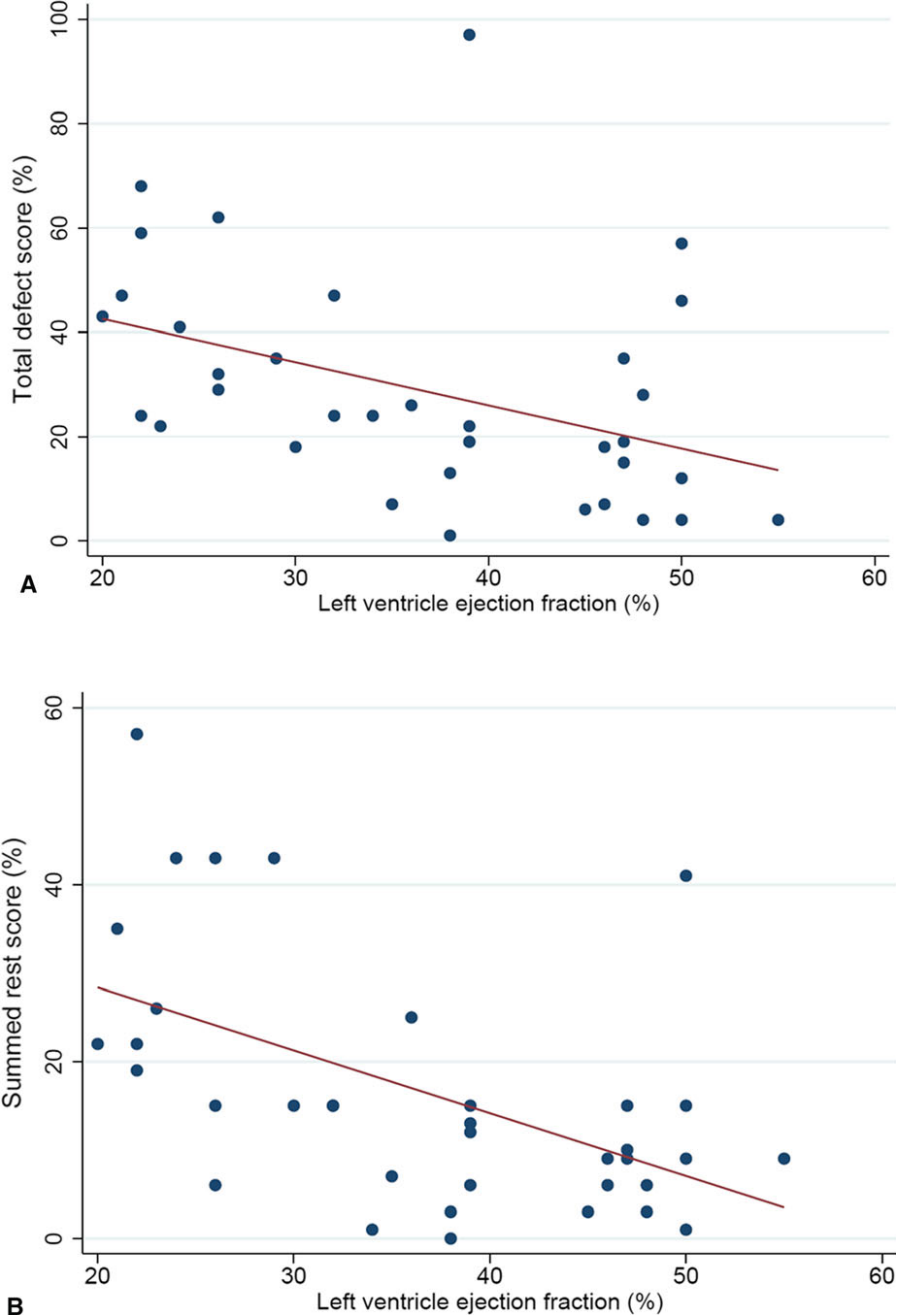
(A) Relationship between left ventricular ejection fraction and innervation defect size. (B) Relationship between left ventricular ejection fraction and perfusion defect size

**Figure 4:**
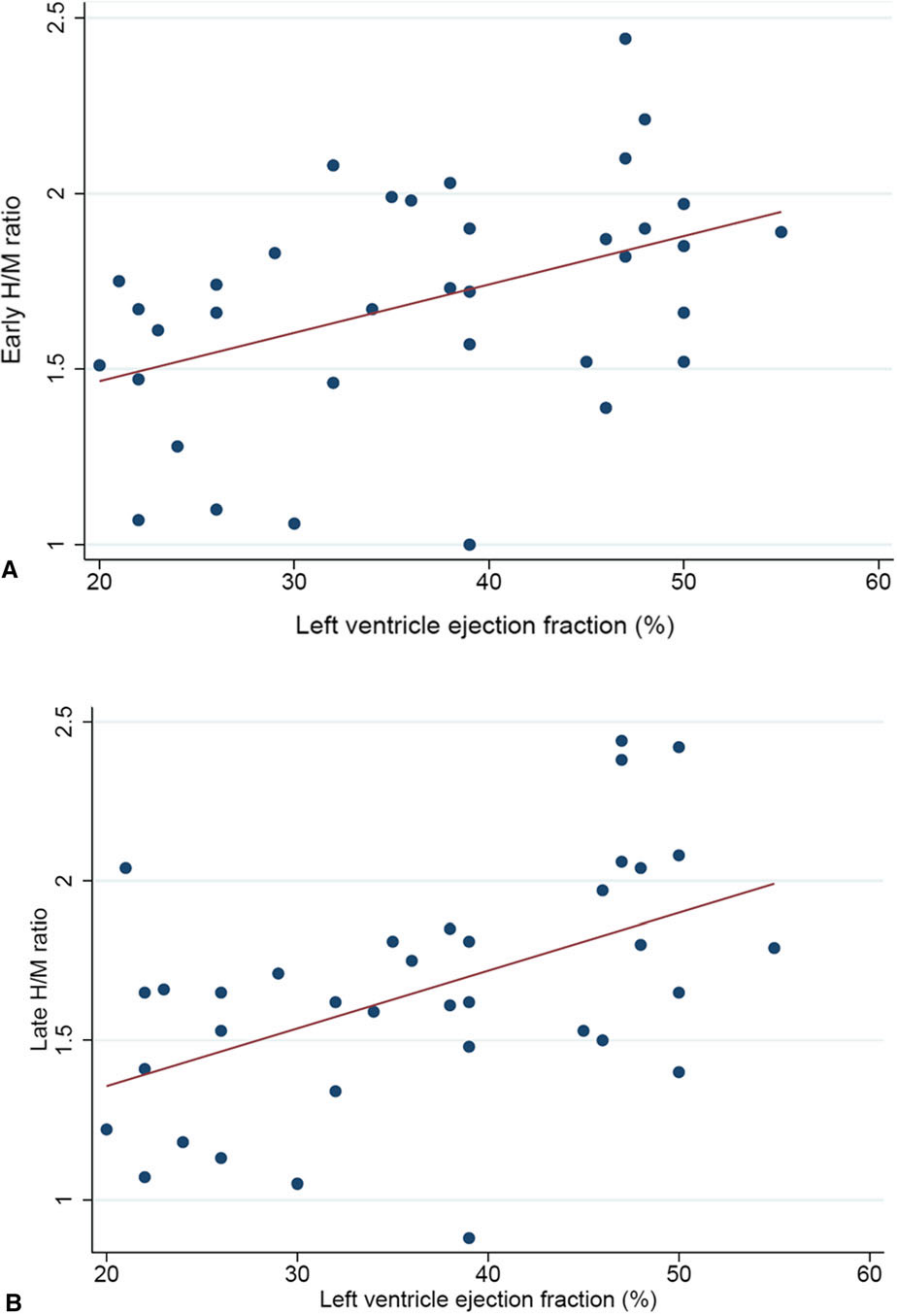
(A) Relationship between left ventricular ejection fraction and early *H*/*M* ratio. (B) Relationship between left ventricular ejection fraction and late *H*/*M* ratio

In the overall study population, both perfusion (*r* = 0.48, *P* < .005) and innervation (*r* = 0.48, *P* < .005) defect sizes correlated with LVEF (Figure 3). A significant correlation of both early (*r* = 0.42, *P* < .05) and late (*r* = 0.47, *P* < .01) H/M ratio with LVEF was also found (Figure 4). Univariable and multivariable logistic regression analyses with reduced LVEF as dependent variable are reported in Table 4. At multivariable analysis, LV hypertrophy and late H/M ratio were independently associated with reduced LVEF.

**Table 4:** Logistic regression analysis with reduced left ventricular ejection fraction as dependent variable

	Univariable		Multivariable	
	Odds ratio (95% CI)	*P* value	Odds ratio (95% CI)	*P* value
Age	1.009 (0.939–1.084)	.81		
Diabetes	0.786 (0.201–3.071)	.72		
Dyslipidemia	0.524 (0.065–4.241)	.52		
Smoking history	1.771 (0.414–7.581)	.45		
Family history of CAD	0.327 (0.071–1.508)	.15		
Known CAD	0.474 (0.096–2.339)	.35		
Symptoms	2.063 (0.492–8.654)	.32		
LV hypertrophy	0.123 (0.026–0.575)	< .01	0.018 (0.001–0.351)	< .05
Perfusion defect size	1.066 (1.005–1.131)	< .05	1.051 (0.950–1.162)	.34
Innervation defect size	1.057 (0.999–1.119)	.06		
Late H/M ratio	0.011 (0.000–0.297)	< .05	0.000 (0.000–0.136)	< .05
Washout rate	1.033 (0.994–1.073)	.09		
Perfusion/innervation mismatch area	1.017 (0.969–1.067)	.49		

*CI*, confidence interval; *CAD*, coronary artery disease; *LV*, left ventricular; *H/M* heart-to-mediastinum.

### Predictors of perfusion/innervation mismatch

**Figure 5:**
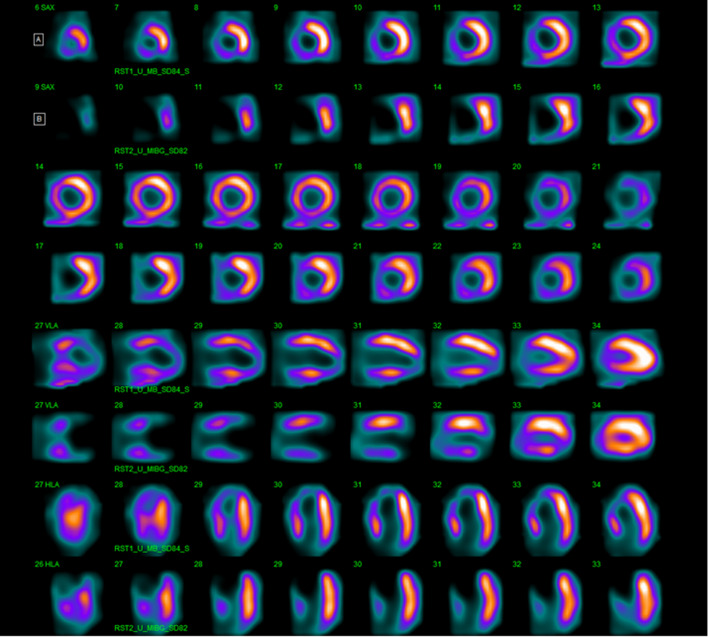
^99m^Tc-sestamibi [A] and ^123^I-MIBG [B] CZT images of a patient with HF and preserved left ventricular ejection fraction (50%). An extensive area of reduced innervation but partially preserved perfusion in apex and antero-septal wall of left ventricle (mismatched area 28%) was visible

**Figure 6:**
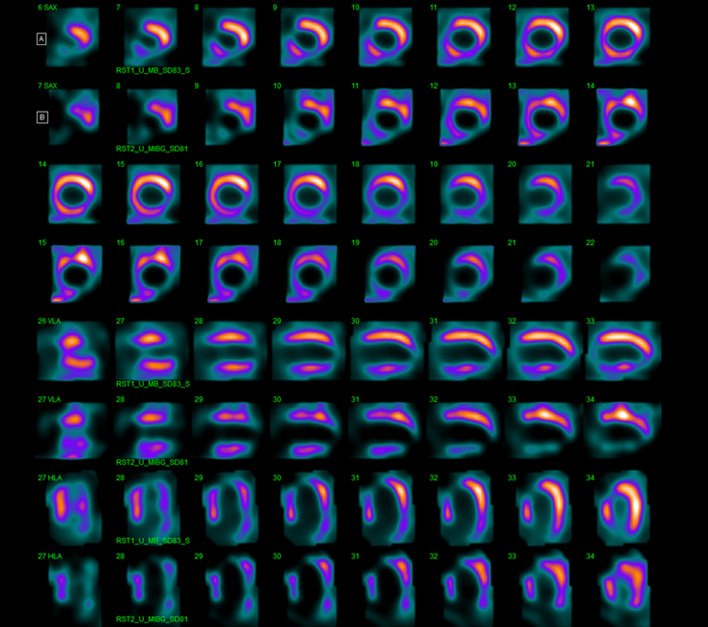
^99m^Tc-sestamibi [A] and ^123^I-MIBG [B] CZT images of a patient with HF and reduced left ventricular ejection fraction (36%). An extensive area of reduced innervation but partially preserved perfusion in apex, antero-septal wall and infero-lateral wall of left ventricle (mismatched area 13%) was visible

In the overall patient population, although perfusion defect size was significantly correlated with innervation defect size (*r* = .73, *P* < .001), innervation defect size was larger than perfusion defect size (29% ± 17% vs. 17% ± 16%, *P* < .001), both in the presence of reduced (33% ± 17% vs. 22% ± 11%, *P* < .005) or mid-range (21% ± 14% vs. 9% ± 12%, *P* < .005) LVEF. Linear regression analyses, considering perfusion/innervation mismatch as dependent variable, are reported in Table 5. At multivariable analysis WR was independently related to mismatch. Images of representative examples of mismatch in a patient with mid-range LVEF and in a patient with reduced LVEF are depicted in Figures 5 and 6.

**Table 5:** Linear regression analysis with perfusion/innervation mismatch area as dependent variable

	Univariable		Multivariable	
	*β* Coefficient (SE)	*P* value	*β* Coefficient (SE)	*P* value
Age	0.275 (0.246)	.27		
Diabetes	6.111 (4.645)	.20		
Dyslipidemia	1.156 (7.573)	.88		
Smoking history	− 0.596 (5.168)	.90		
Family history of CAD	− 9.805 (4.585)	.05		
Known CAD	6.304 (5.624)	.27		
Symptoms	4.876 (4.756)	.31		
LV hypertrophy	− 3.143 (4.854)	.52		
Late H/M ratio	− 9.802 (6.215)	.12		
Washout rate	0.189 (0.081)	< .05	0.189 (0.081)	< .05
LV ejection fraction	0.295 (0.223)	.19		

*SE*, standard error; *CAD*, coronary artery disease; *LV*, left ventricular; *H/M*, heart-to-mediastinum.

## Discussion

To our knowledge this is the first study assessing the feasibility of a low-dose SDI ^123^I/^99m^Tc single imaging session protocol in patients with HF using CZT-SPECT. It is already known that cardiac CZT-SPECT systems have a twofold improvement in energy resolution, allowing simultaneous dual-isotope acquisition with lower down-scatter of the two isotope photo-peaks as compared to conventional SPECT.^15,16^ When used in combination, the close photo-peaks of ^99m^Tc- and ^123^I-isotopes require careful evaluation to discriminate their energy properties while maintaining count sensitivity. It has been demonstrated using CZT scanners that excellent concordance between ^123^I-/^99m^Tc-labeled dual- and single-images was achieved, in both phantom and animal studies, thanks to high spatial and energy resolution of the CZT systems, thus allowing count sensitivity to be maintained.^17,18^ The simultaneous use of ^123^I and ^99m^Tc could lead to a reduction in total procedure time with good quality images, improved patient comfort and increased throughput, with the advantage of obtaining both innervation and perfusion parameters from the same exam session. ^123^I-MIBG SPECT images can be compared with SPECT myocardial perfusion images to examine differences between regional innervation and perfusion. In making such comparisons, it is important to be aware of differences between tracer distribution in normal subjects as compared to pathological innervation and perfusion patterns, such as lower uptake of ^123^I-MIBG seen in the posterior inferior wall using a traditional camera.^19,20^ This study evaluating the tracer distribution in a control group confirms that a lower uptake in the inferior wall is a physiologic pattern also using a CZT-SPECT camera.

In addition, simultaneous perfusion and sympathetic innervation imaging enables the evaluation of perfusion/innervation mismatch and may provide valuable information to target the trigger zone in the setting of ventricular arrhythmia.^21^ Gimelli et al^22^ using a sequential ^123^I-MIBG and ^99m^Tc-tetrofosmin CZT acquisition demonstrated a correlation between impaired myocardial innervation and systolic LV function. In particular, using a sequential protocol with 74-111 MBq of ^123^I-MIBG for innervation imaging and 222-259 MBq of ^99m^Tc-tetrofosmin for perfusion scans^22^ in 28 patients with and without ischemic heart disease, the authors showed that alteration of early innervation parameters was significantly correlated with impaired myocardial perfusion and LV dysfunction.

Moreover, using a low-dose SDI protocol, we were able to confirm a relationship between myocardial innervation, perfusion parameters and LV systolic function. Patients with reduced LVEF showed higher values of perfusion and innervation defect sizes and WR, and lower values of early and late *H*/*M* ratio compared to patients with preserved or mid-range LVEF. Moreover, in our study population LV hypertrophy and late *H*/*M* ratio were independently associated with reduced LVEF. It has been demonstrated that optimal mapping of regional myocardial sympathetic denervation is obtained with delayed acquisition.^23^ Therefore, for the semiquantitative analysis of innervation, we considered the delayed images. For our analysis we used no scattered correction values, as it has been demonstrated that ^99m^Tc crosstalk into the ^123^I window is negligible when performing a simultaneous CZT-SPECT acquisition.^9,24^ We also assessed myocardial perfusion/innervation mismatch and although perfusion defect size was correlated with innervation defect size, ^123^I-MIBG defect size was significantly larger than perfusion defects in both groups of patients with preserved or mid-range LVEF and reduced LVEF. These findings indicate that independently of LVEF, a larger denervation area is always present around an area of myocardial hypoperfusion or necrosis area. This is in line with notion that these sympathetic nerve endings are more prone to ischemic damage compared to cardiomyocytes. In addition, WR significantly correlated with perfusion/innervation mismatch. It has been suggested that WR reflects neuronal integrity of sympathetic tone/drive mainly representing uptake-1 (i.e., NET: norepinephrine transporter).^13^ Hence, WR correlates with the more extensive involvement of neuronal retention system in the mismatch area characterized by a higher amount of adrenergic denervation compared to perfusion impairment. As demonstrated in previous studies, ^123^I-MIBG WR and late *H*/*M* ratio have a prognostic role in patients with HF.^25^ In particular, a high WR represents an independent predictor for subsequent cardiac events, although lack of correlation to LV function.^26^ The measurement of perfusion/innervation mismatch, as index of regions with impaired sympathetic innervation and preserved viability and frequently localized at the border of infarcted areas, is a key step in prognosis assessment, as it has been demonstrated that these mismatch areas are triggers of ventricular arrythmias and may be used as potential therapeutic targets.^27^ Therefore, markers of cardiac sympathetic impairment obtained by CTZ-SPECT could be used to evaluate the clinical and prognostic role of denervated but viable myocardium, improving the risk stratification of high-risk patients. Resting myocardial perfusion imaging is an imaging tool to detect viable myocardium in patients with LV dysfunction with prognostic information incremental to that of clinical data and LV function.^28–30^

One of the strengths of the present study is represented by the use of a simultaneous protocol with very low dose injected activities (i.e., 74 MBq of ^123^I-MIBG and 185 MBq of ^99m^Tc-sestamibi) leading to a radiation dose reduction of 31% compared to a standard protocol (from 3.0 to 4.4 mSv, respectively) without losing in accuracy. This is particularly relevant considering that reduction in radiopharmaceutical dosage enables more cost effective nuclear myocardial perfusion imaging, a very welcome trend against the background of continually increasing use of nuclear cardiology in an increasingly challenging fiscal environment with ever-rising healthcare costs.^31^ Potential cost reduction in conjunction with the lower exposure could also lead to a marked improvement for dual-isotope acquisition imaging in the evaluation of patient with heart failure.

It must be also considered that patients with HF impaired myocardial innervation may have low myocardial ^123^I-MIBG uptake with suboptimal localization of the heart, therefore requiring a dual-isotope protocol to localize the heart.^6^ An SDI protocol using perfusion images to define heart contouring enables more precise measurement of ^123^I-MIBG heart uptake, thus achieving a higher level of interpreted data accuracy, as well as enabling perfect co-registration of innervation and perfusion studies, with an excellent assessment of innervation-perfusion mismatch.

## New knowledge gained

Low-dose CZT-SPECT cardiac imaging may enable rapid assessment of differences in LV perfusion and innervation patterns, possibly better characterizing cardiac functional status with reduced radiation exposure and shorter examination time and an associated quality improvement over single isotope studies.

## Conclusions

The extent of both myocardial innervation and perfusion defects are related to a reduction of LV systolic function, and late *H*/*M* ratio result was an independent predictor of reduced LVEF. Among parameters of cardiac innervation, WR results significantly correlated to extent of perfusion/innervation mismatch area and this could have important clinical implications.

## Supplementary Information

Below is the link to the electronic supplementary material.

Supplementary file1 (PPTX 2311 KB)

Supplementary file2 (PDF 332 KB)

Supplementary file3 (MP3 7143 KB)

## Abbreviations

MIBG: Metaiodobenzylguanidine
HF: Heart failure
H/M: Heart-to-mediastinum
WR: Washout rate
SPECT: Single-photon emission computed tomography
CZT: Cadmium–zinc–telluride
SDI: Simultaneous dual-isotope
LV: Left ventricular
EF: Ejection fraction
ROI: Region of interest
