# Supplementary material for: Simultaneous assessment of myocardial perfusion and adrenergic innervation in patients with heart failure by low-dose dual-isotope CZT SPECT imaging
Source: J Nucl Cardiol. 2022 Apr 4;29(6):3341–51. doi: 10.1007/s12350-022-02951-4 (PMC9834348; doi:10.1007/s12350-022-02951-4)
Supplement: Supplementary file 1 — Supplementary file1 (PPTX 2311 KB) [file 12350_2022_2951_MOESM1_ESM.pptx]

## Slide 1
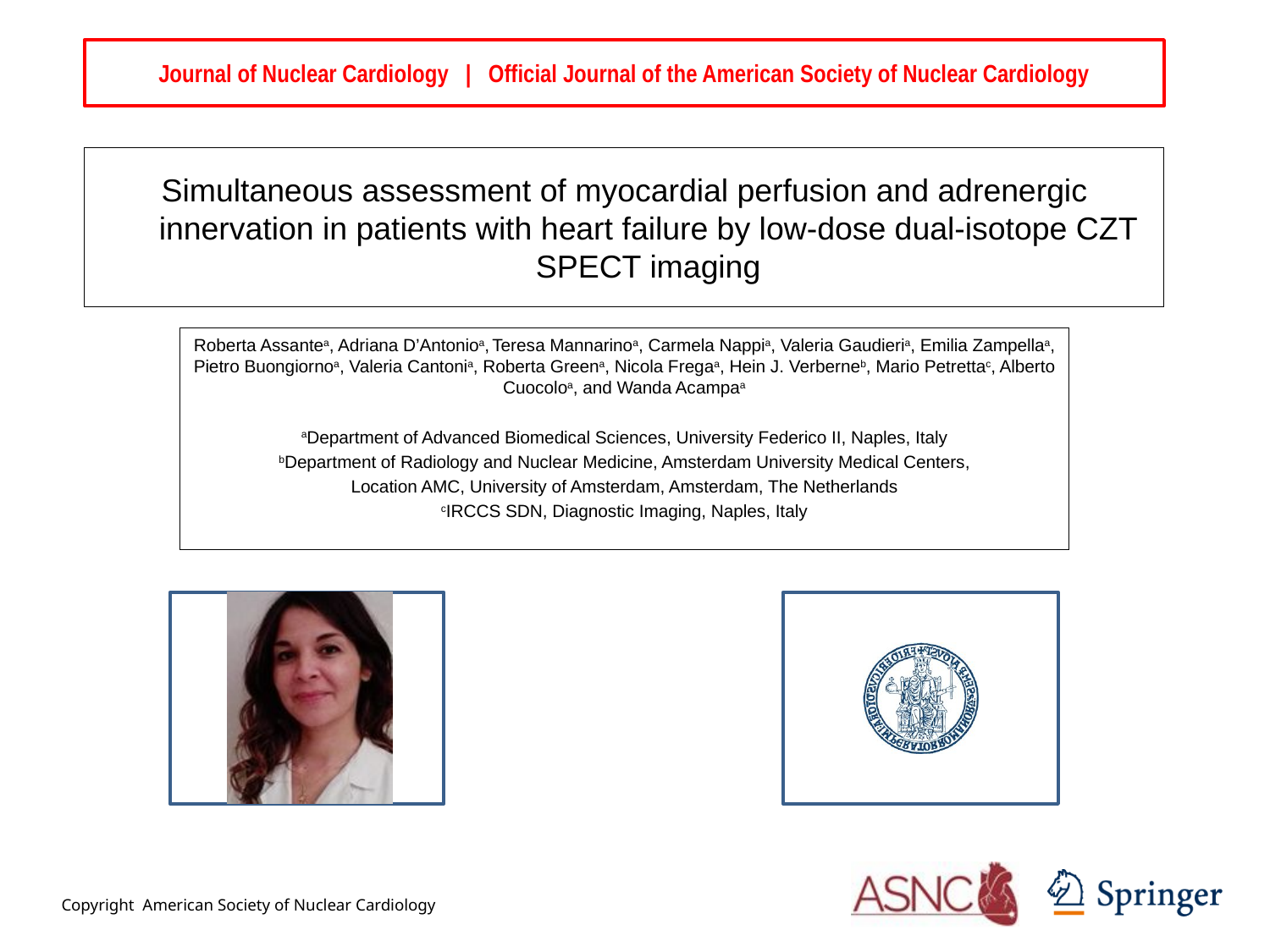

Journal of Nuclear Cardiology | Official Journal of the American Society of Nuclear Cardiology
# Simultaneous assessment of myocardial perfusion and adrenergic innervation in patients with heart failure by low-dose dual-isotope CZT SPECT imaging
Roberta Assantea, Adriana D’Antonioa, Teresa Mannarinoa, Carmela Nappia, Valeria Gaudieria, Emilia Zampellaa, Pietro Buongiornoa, Valeria Cantonia, Roberta Greena, Nicola Fregaa, Hein J. Verberneb, Mario Petrettac, Alberto Cuocoloa, and Wanda Acampaa
aDepartment of Advanced Biomedical Sciences, University Federico II, Naples, Italy
bDepartment of Radiology and Nuclear Medicine, Amsterdam University Medical Centers,
Location AMC, University of Amsterdam, Amsterdam, The Netherlands
cIRCCS SDN, Diagnostic Imaging, Naples, Italy
Copyright American Society of Nuclear Cardiology

## Slide 2
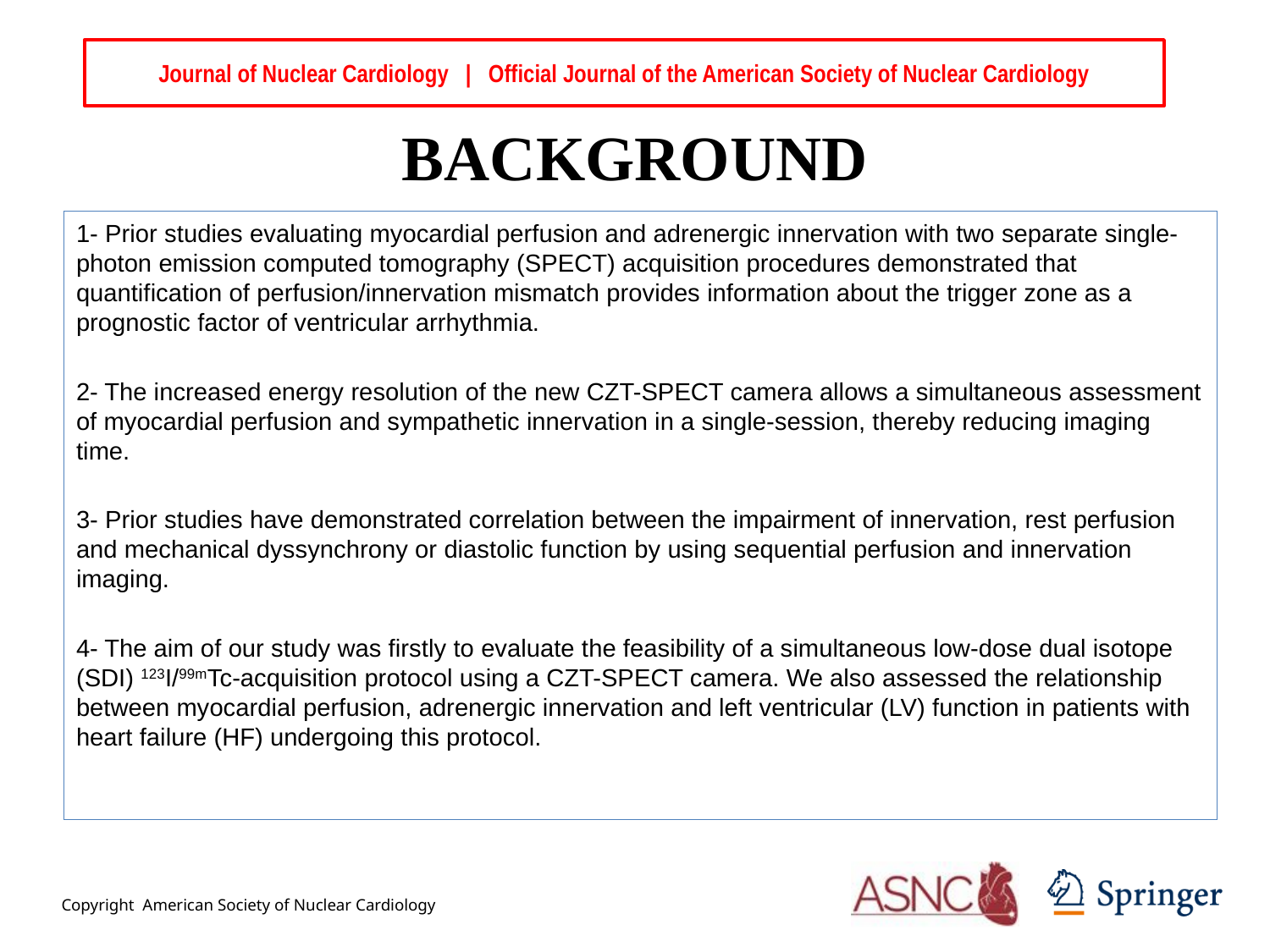

Journal of Nuclear Cardiology | Official Journal of the American Society of Nuclear Cardiology
# BACKGROUND
1- Prior studies evaluating myocardial perfusion and adrenergic innervation with two separate single-photon emission computed tomography (SPECT) acquisition procedures demonstrated that quantification of perfusion/innervation mismatch provides information about the trigger zone as a prognostic factor of ventricular arrhythmia.
2- The increased energy resolution of the new CZT-SPECT camera allows a simultaneous assessment of myocardial perfusion and sympathetic innervation in a single-session, thereby reducing imaging time.
3- Prior studies have demonstrated correlation between the impairment of innervation, rest perfusion and mechanical dyssynchrony or diastolic function by using sequential perfusion and innervation imaging.
4- The aim of our study was firstly to evaluate the feasibility of a simultaneous low-dose dual isotope (SDI) 123I/99mTc-acquisition protocol using a CZT-SPECT camera. We also assessed the relationship between myocardial perfusion, adrenergic innervation and left ventricular (LV) function in patients with heart failure (HF) undergoing this protocol.
Copyright American Society of Nuclear Cardiology

## Slide 3
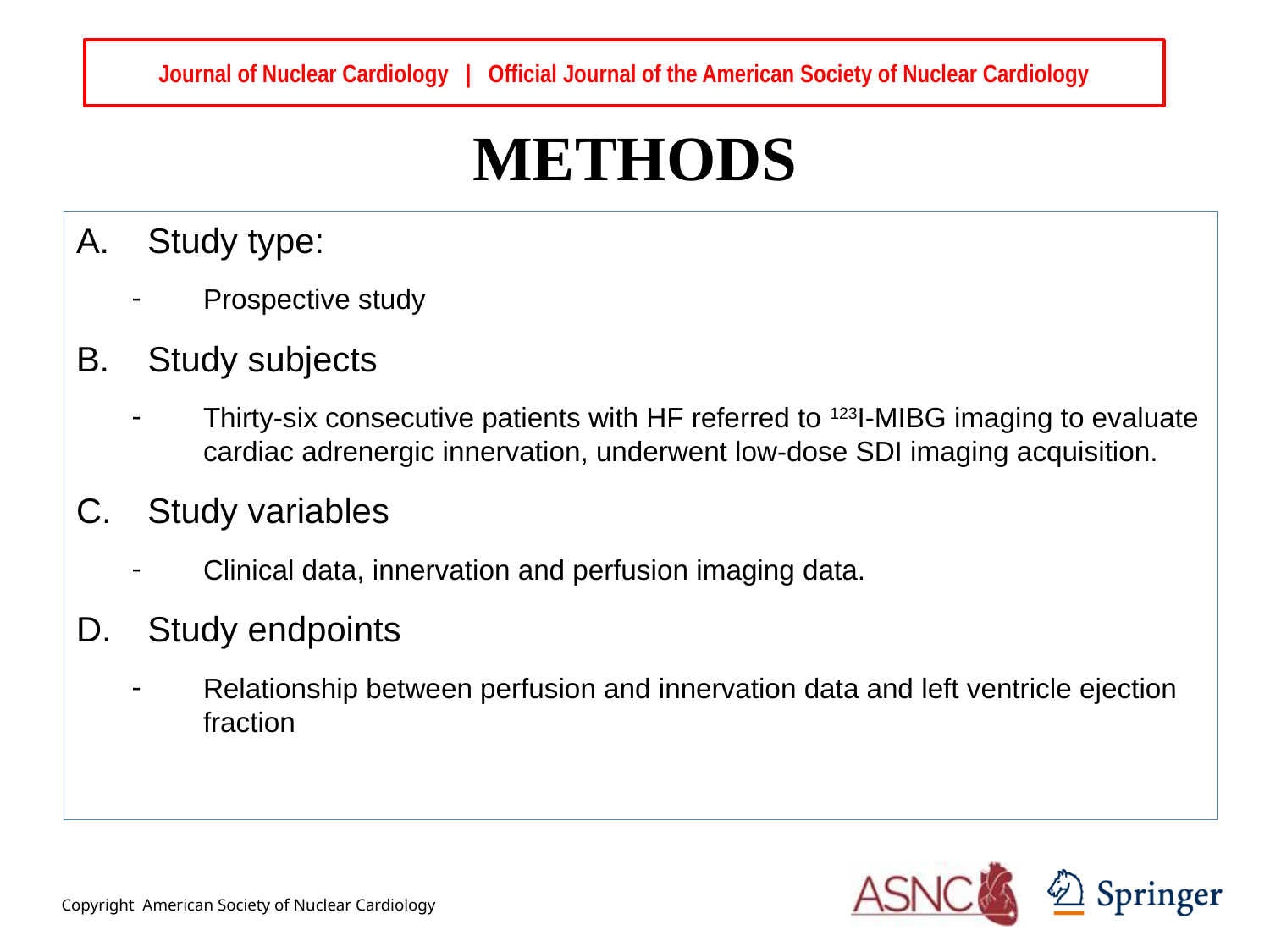

Journal of Nuclear Cardiology | Official Journal of the American Society of Nuclear Cardiology
# METHODS
Study type:
Prospective study
Study subjects
Thirty-six consecutive patients with HF referred to 123I-MIBG imaging to evaluate cardiac adrenergic innervation, underwent low-dose SDI imaging acquisition.
Study variables
Clinical data, innervation and perfusion imaging data.
Study endpoints
Relationship between perfusion and innervation data and left ventricle ejection fraction
Copyright American Society of Nuclear Cardiology

## Slide 4
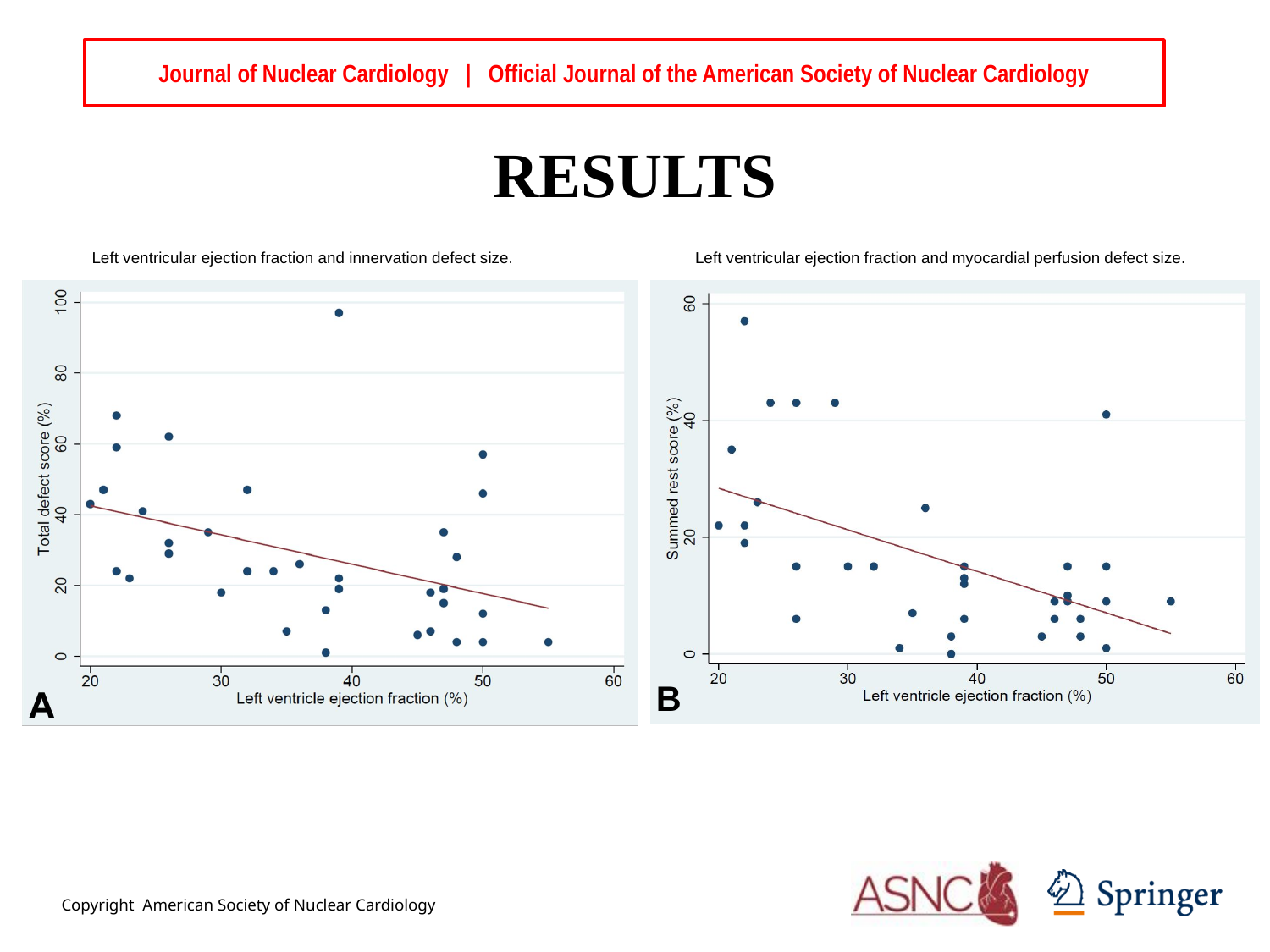

Journal of Nuclear Cardiology | Official Journal of the American Society of Nuclear Cardiology
# RESULTS
Left ventricular ejection fraction and myocardial perfusion defect size.
Left ventricular ejection fraction and innervation defect size.
Copyright American Society of Nuclear Cardiology

## Slide 5
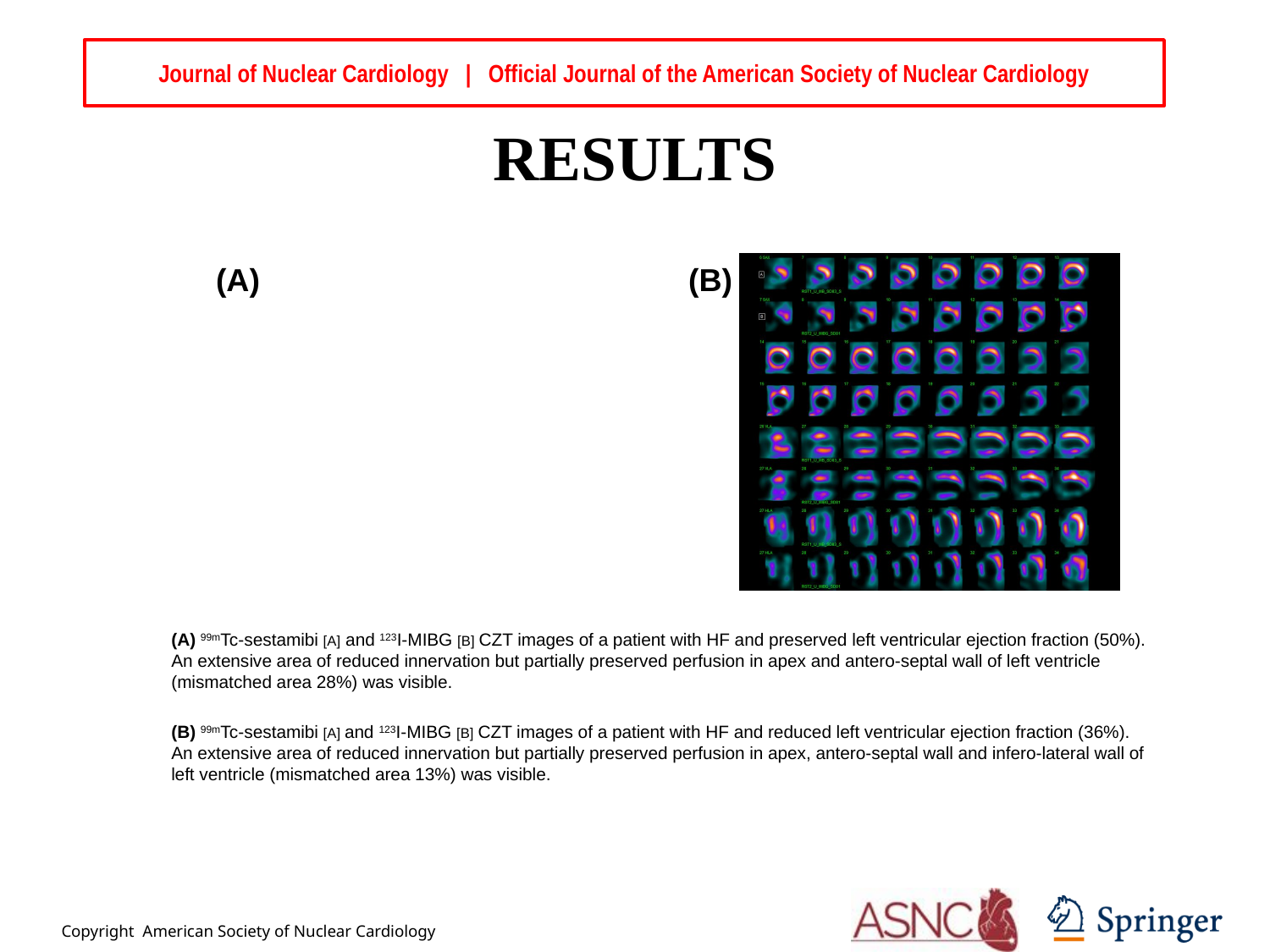

Journal of Nuclear Cardiology | Official Journal of the American Society of Nuclear Cardiology
# RESULTS
(A)
(B)
(A) 99mTc-sestamibi [A] and 123I-MIBG [B] CZT images of a patient with HF and preserved left ventricular ejection fraction (50%). An extensive area of reduced innervation but partially preserved perfusion in apex and antero-septal wall of left ventricle (mismatched area 28%) was visible.
(B) 99mTc-sestamibi [A] and 123I-MIBG [B] CZT images of a patient with HF and reduced left ventricular ejection fraction (36%). An extensive area of reduced innervation but partially preserved perfusion in apex, antero-septal wall and infero-lateral wall of left ventricle (mismatched area 13%) was visible.
Copyright American Society of Nuclear Cardiology

## Slide 6
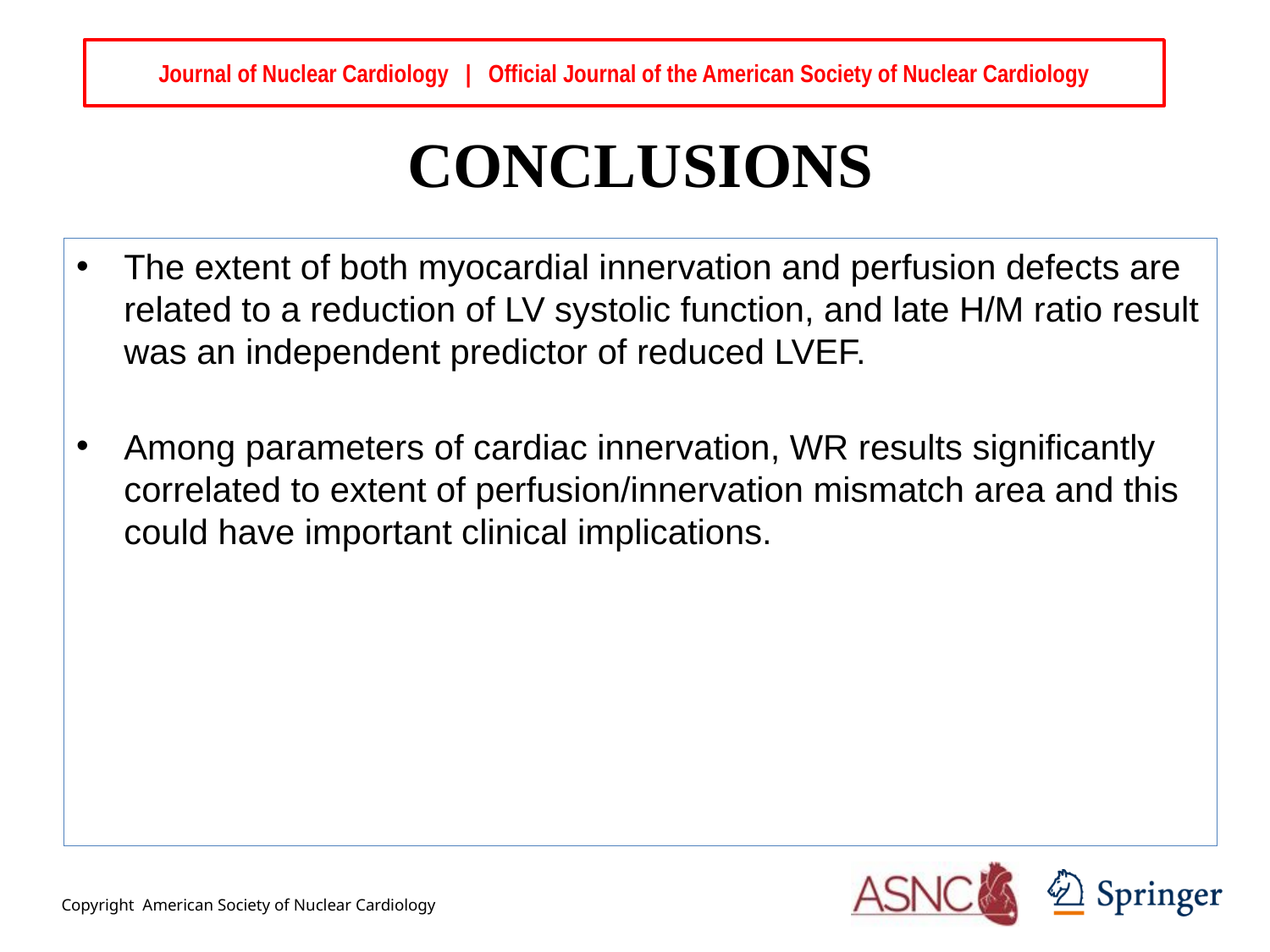

Journal of Nuclear Cardiology | Official Journal of the American Society of Nuclear Cardiology
# CONCLUSIONS
The extent of both myocardial innervation and perfusion defects are related to a reduction of LV systolic function, and late H/M ratio result was an independent predictor of reduced LVEF.
Among parameters of cardiac innervation, WR results significantly correlated to extent of perfusion/innervation mismatch area and this could have important clinical implications.
Copyright American Society of Nuclear Cardiology
