# Supplementary material for: Simultaneous assessment of myocardial perfusion and adrenergic innervation in patients with heart failure by low-dose dual-isotope CZT SPECT imaging
Source: J Nucl Cardiol. 2022 Apr 4;29(6):3341–51. doi: 10.1007/s12350-022-02951-4 (PMC9834348; doi:10.1007/s12350-022-02951-4)
Supplement: Supplementary file 2 — Supplementary file2 (PDF 332 KB) [file 12350_2022_2951_MOESM2_ESM.pdf]

(A)

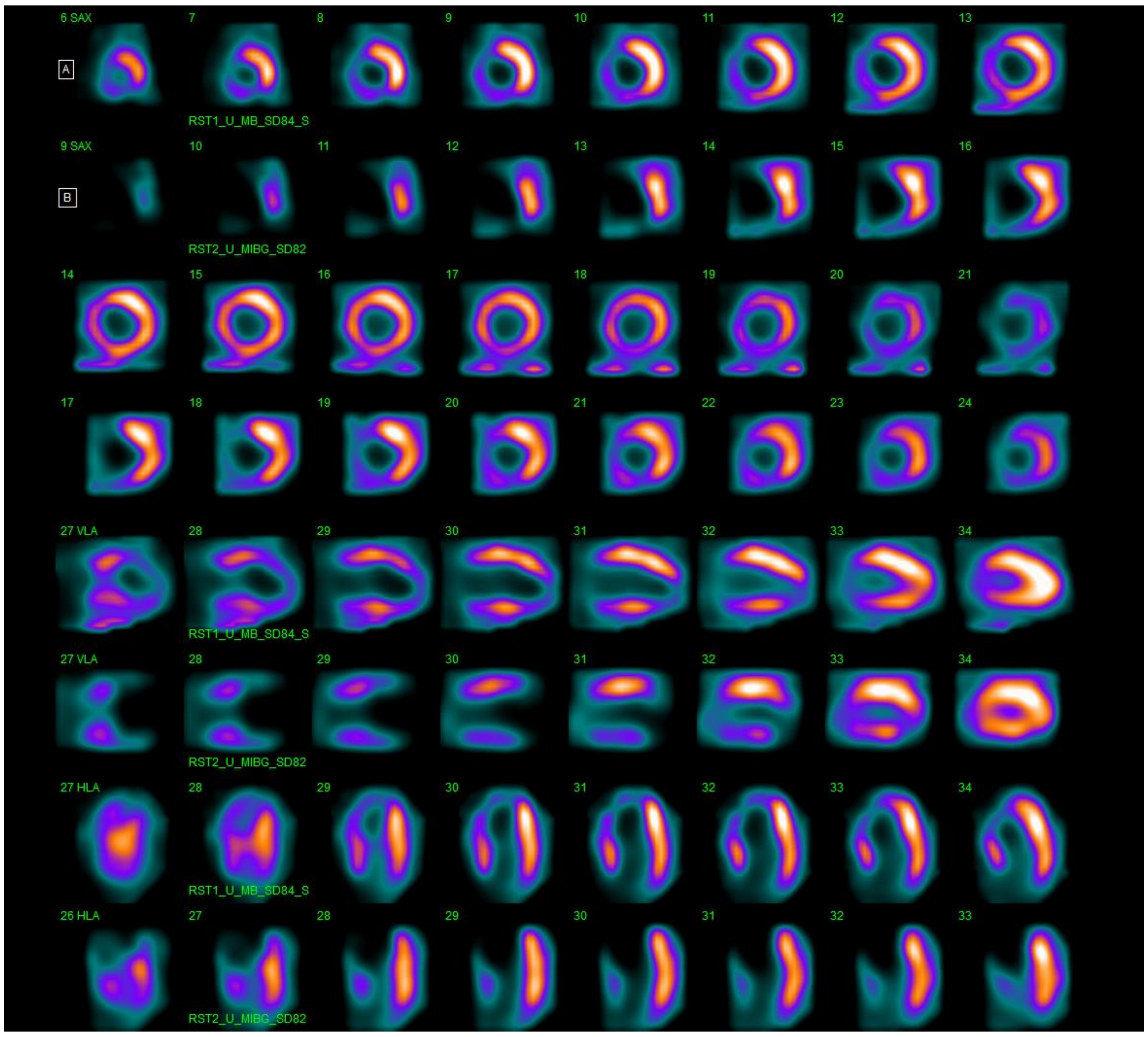

(B)

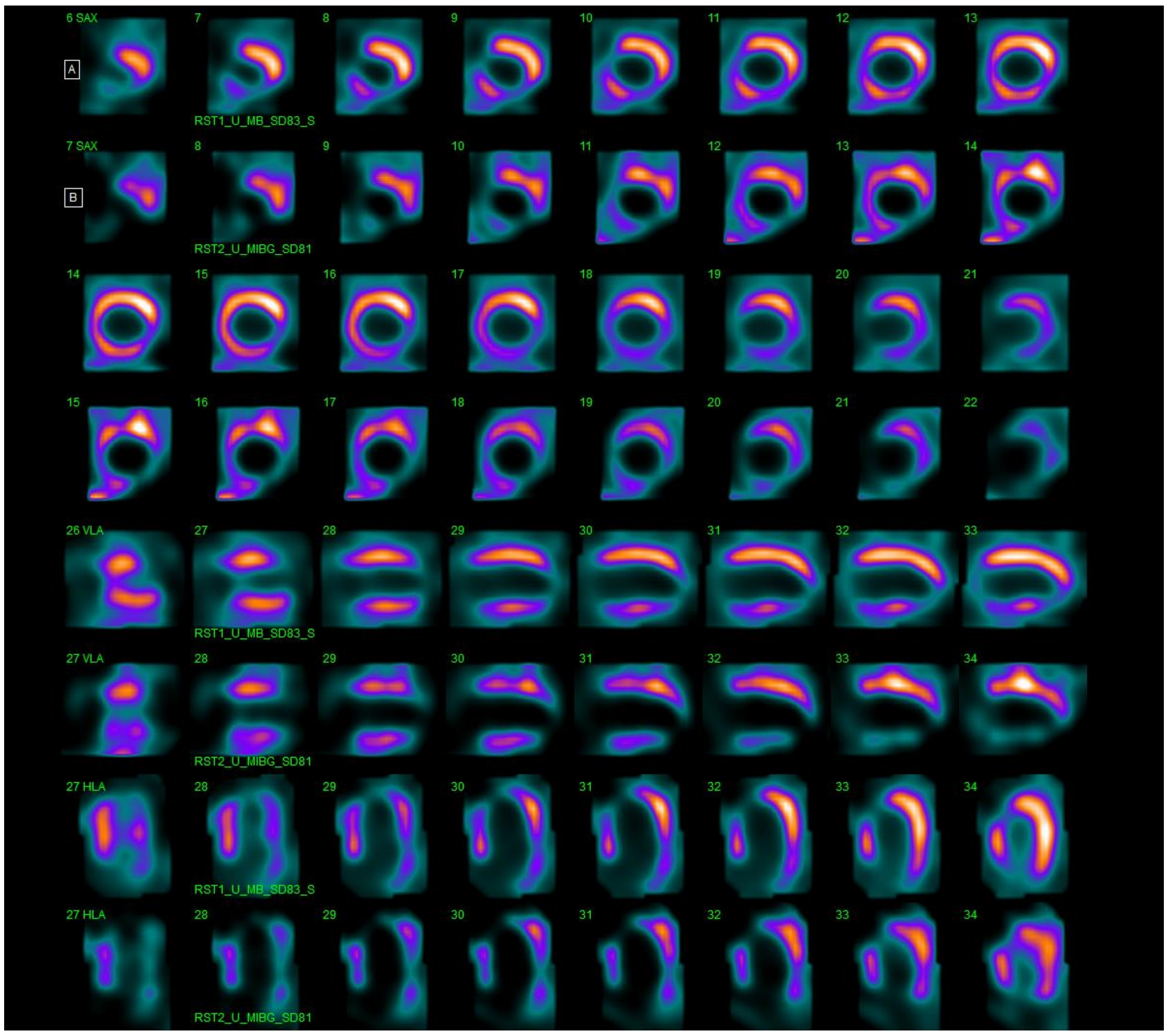

(A) ^{99m}Tc-sestamibi [A] and 123I-MIBG [B] CZT images of a patient with HF and preserved left ventricular ejection fraction (50%). An extensive area of reduced innervation but partially preserved perfusion in apex and antero-septal wall of left ventricle (mismatched area 28%) was visible. **(B)** ^{99m}Tc-sestamibi [A] and ^{123}I-MIBG [B] CZT images of a patient with HF and reduced left ventricular ejection fraction (36%). An extensive area of reduced innervation but partially preserved perfusion in apex, antero-septal wall and infero-lateral wall of left ventricle (mismatched area 13%) was visible.
